# Prognostic Value of Serum Transferrin Analysis in Patients with Ovarian Cancer and Cancer-Related Functional Iron Deficiency: A Retrospective Case–Control Study

**DOI:** 10.3390/jcm11247377

**Published:** 2022-12-12

**Authors:** Tatiana I. Ivanova, Ilya D. Klabukov, Ludmila I. Krikunova, Marina V. Poluektova, Natalia I. Sychenkova, Vera A. Khorokhorina, Nikolay V. Vorobyev, Margarita Ya. Gaas, Denis S. Baranovskii, Oksana S. Goryainova, Anastasiya M. Sachko, Peter V. Shegay, Andrey D. Kaprin, Sergei V. Tillib

**Affiliations:** 1 A. Tsyb Medical Radiological Research Center—Branch of the National Medical Research Radiological Center of the Ministry of Health of the Russian Federation, Zhukova Str. 10, 249030 Obninsk, Russia; 2 Institute of Gene Biology of the Russian Academy of Sciences, Vavilova Str. 34/5, 119334 Moscow, Russia; 3 National Medical Research Radiological Center of the Ministry of Health of the Russian Federation, Koroleva Str. 4, 249036 Obninsk, Russia; 4 Department of Urology and Operative Nephrology, Peoples’ Friendship University of Russia (RUDN University), Miklukho-Maklaya Str. 6, 117198 Moscow, Russia; 5 Obninsk Institute for Nuclear Power Engineering, National Research Nuclear University MEPhI, Studgorodok 1, 249039 Obninsk, Russia; 6 Department of Oncology, Radiotherapy and Plastic Surgery, Sechenov First Moscow State Medical University (Sechenov University), Trubetskaya str. 8-2, 119991 Moscow, Russia; 7 P.A. Hertsen Moscow Oncology Research Institute—Branch of the National Medical Research Radiological Center of the Ministry of Health of the Russian Federation, 2nd Botkinsky Proezd 3, 125284 Moscow, Russia

**Keywords:** biomarker, cancer, functional iron deficiency, monoclonal nanobodies, holo-transferrin, apo-transferrin, transferrin, ovarian cancer

## Abstract

(1) Background: There are no reliable and widely available markers of functional iron deficiency (FID) in cancer. The aim of the study was to evaluate the role of transferrin (Tf) as a marker of cancer of the ovary (CrO) and related FID. (2) Methods: The study groups consisted of 118 patients with CrO and 69 control females. Blood serum iron status was determined on a Beckman Coulter AU (USA) analyzer. Tf quantification was performed by immunoturbidimetry. The relative contents of apo- and holo-Tf (iron-free and iron-saturated Tf, respectively) were determined in eight patients and a control female by immunochromatographic analysis based on the use of monoclonal single-domain antibodies (nanobodies). (3) Results: Four groups of patients with different iron statuses were selected according to ferritin and transferrin saturation values: absolute iron deficiency (AID) (*n* = 42), FID (*n* = 70), iron overload (*n* = 4), normal iron status (*n* = 2). The groups differed significantly in Tf values (*p* < 0.0001). Lower values of Tf were associated with FID. Furthermore, FID is already found in the initial stages of CrO (26%). Immunosorbents based on nanobodies revealed the accumulation of apo-Tf and the decrease in holo-Tf in patients with CrO. (4) Conclusions: Tf may be a promising tool for diagnosing both CrO and associated FID.

## 1. Introduction

Cancer of the ovary (CrO) is the most common cause of death among oncological diseases of the female reproductive system [1]. Deaths from ovarian cancer account for 47% of all deaths from cancer of the female reproductive system [2]. In 75% of cases, CrO is diagnosed at stages III–IV, so the prognosis is extremely unfavorable. Methods for screening the population for CrO have not been developed, so the search for potential markers and therapeutic targets is an urgent task [3]. Iron is an important component of proteins and enzymes that perform a wide range of functions, such as cellular respiration, oxygen transport and metabolism, energy metabolism, DNA synthesis and repair, signal transduction, and immune response. At the same time, iron is a very reactive element, the excess of which induces oxidative stress with the formation of reactive oxygen species and products of lipid peroxidation. This fact underlies the recently discovered iron-dependent type of cell death, ferroptosis, which represents great prospects for the treatment of malignant tumors [4,5,6].

The maintenance and regulation of iron homeostasis are carried out by a cascade of proteins, where the failure of one of the links may lead to severe pathological complications. Iron transport and deposition are carried out mainly through iron-transferrin and iron-ferritin complexes. Epidemiological studies have found associations between altered iron metabolism and cancer [7,8,9,10]. Iron deficiency is known to cause the recurrence of estrogen-dependent cancers in young and middle-aged women, while iron overload stimulates carcinogenesis in elderly women [11]. At the time of malignant disease diagnosis, patients can have either functional or absolute iron deficiency (FID or AID, respectively). FID is the most common and is characterized by a condition where the total amount of iron may be normal or even elevated due to transport and recirculation disorders that lead to the accumulation of iron ions in the ferritin [12]. Pathogenesis includes many factors associated with chronic activation of the immune response in malignant processes through the suppressive effect of cytokines (TNF, IL-1, IL-6) on erythropoiesis, with the further development of cancer-related anemia (CRA) [13]. Acute inflammation intensifies as the disease progresses and turns into a state of chronic inflammation. A distinctive feature of CRA is the combination of high ferritin levels (100–800 µg/L) with low serum iron, transferrin saturation (TSAT) (less than 10 µmol/L and 20%, respectively), and low hemoglobin (<120 g/L for females) [4,8,13,14,15,16,17]. However, the TSAT score has limitations in cancer patients and may have inadequately high or false normal values [18].

The overall incidence of CRA in solid tumors is more than 30%, and it strongly increases in the case of a malignant progression (stage III, IV cancer), reaching 68% in women and 77% in men [19]. The frequency of CRA depends on the type of cancer and is more common in malignant diseases of the lung, urogenital and gynecological organs, and the gastrointestinal tract [20]. Prostate cancer is accompanied by CRA in 80% of cases, and ovarian cancer (CrO) is accompanied in 68%. Gender affects the incidence of CRA, so in the case of bladder cancer, it is 81% in men and 65% in women; in lung cancer, it is 87% in men and 63.6% in women [21]. The timely detection of FID in cancer patients and the prevention of CRA are extremely important tasks because, in the course of combined chemotherapy with cytostatics, CRA is the cause of side effects. Special treatment algorithms are necessary in cases of anemia and iron deficiency in patients with cancer [16]. In addition, cytostatics or their combination with radiotherapy often induce CRA [13,15,22,23]. Currently, there is no single reliable and accessible biochemical marker for FID [12]. In the absence of malignant processes and inflammation, plasma ferritin currently seems to be the most sensitive and specific marker for diagnosing the degree of iron deficiency (ID) [24,25]. In cancer patients, high ferritin >100 µg/L in combination with low transferrin saturation (TSAT) (<20%) is suggested as a criterion of FID, and ferritin <100 µg/L is a criterion of absolute iron deficiency (AID) [16]. 

Since ferritin is an acute-phase protein, and its amount increases in the presence of inflammatory disease or cancer, correction factors based on the determination of concentrations of well-studied acute-phase proteins (C-reactive protein (CRP) and α1-acid glycoprotein (AGP)) are introduced to diagnose both iron overload and deficiency. Currently, one of the urgent tasks is to find other acute phase proteins, the level of which could complement and correct the data on ferritin [25,26,27]. The literature discusses the promising use of acute-phase inflammation proteins not only for the diagnosis of iron deficiency anemia and CRA but also for noninvasive personalized cancer diagnosis [28]. In a retrospective study of 427 patients with hepatocellular carcinoma who underwent radical liver resection, serum ferritin was found to be a simple, inexpensive, convenient, and reliable prognostic factor for survival [29]. Elevated serum ferritin levels have been shown to be a biomarker for pancreatic cancer, renal cell carcinoma, lung cancer, and head and neck cancer; moreover, it is a sensitive marker for detecting advanced tumor stages [30]. However, little attention has been paid to other acute-phase iron-related proteins, such as transferrin.

Transferrin (Tf), like ferritin, is a traditional biochemical indicator for diagnosing blood iron homeostasis and is the main plasma protein transporting iron and an acute-phase reactant. Its levels are decreased in inflammation, malignant processes, and iron overload and elevated in iron deficiency [12,31,32]. Recently, it has been shown that reduced serum transferrin levels can be used as a valuable predictor of serious side effects and disease progression in COVID-19 [33]. Shigeta et al. demonstrated a stimulating role of transferrin in the development of advanced serous ovarian cancer [34]. Based on preliminary results using proteomic methods with polyclonal antibodies to Tf, it was stated that Tf may be a useful biomarker for ovarian cancer diagnosis and treatment [35]. Tf was included in the OVA 1 panel approved by the Food and Drug Administration (FDA) as a risk-of-ovarian-cancer algorithm [36]. The role of transferrin in the delivery of metal-containing anticancer drugs is being investigated [37,38,39].

The detectable concentration of iron in serum is predominantly related to Fe(III) ions bound to serum transferrin. Each transferrin molecule is capable of binding two Fe(III) ions. In biological fluids, transferrin predominantly exists as iron-saturated (holo-Tf) and iron-unsaturated (apo-Tf) forms of the protein. To date, its role in the pathogenesis of CrO and CRA has not been sufficiently studied. For transferrin, we could not find threshold values for different pathologies of iron metabolism in malignant diseases. 

For the direct analysis of changes in the relative representation of different forms of transferrin potentially associated with a pathological process, specific antibodies can be used. Recently, the authors of this study were able to obtain and characterize highly specific single-domain antibodies (nanobodies) recognizing only holo-Tf (aTf1) or apo-Tf (aTf2) [40]. It was shown that the use of immunosorbents based on these nanobodies can detect changes in the relative representation of apo-Tf and holo-Tf in human biological fluids [40]. In this work, we used these immunosorbents and selected immunoaffinity fractionation conditions to directly analyze the ratio of holo-Tf to apo-Tf in the blood of patients with ovarian cancer and cancer-free females.

The aim of our study was to assess the role of transferrin in the prognosis of cancer of the ovary and related iron deficiency.

## 2. Materials and Methods

### 2.1. Study Design

We performed an observational, retrospective case-control study. CrO groups were composed of women admitted to the A. Tsyb Medical Radiological Research Center—branch of the National Medical Research Radiological Center of the Russian Federation (MRRC)—between May 2011 and May 2021 with newly diagnosed CrO. The control group included volunteer cancer-free females recruited among employees of the MRRC and examined in the course of routine medical examinations performed by MRRC physicians. Blood samples from cancer-free females were collected in the same period. Females in the study and control groups were residents of small towns (with 30–100 thousand inhabitants) in central European Russia, mainly the Kaluga Region. A total of 201 females were recruited in the study. Table S1 lists patients and controls data enrolled in the study.

### 2.2. Participants Selection 

The exclusion criteria for both the study and control groups consisted of a lack of written informed consent, a previous history of any type of cancer, plasma with hemolyzed red blood cells, and hematologic, hepatic, or renal diseases. For the control group, individuals under 30 years of age were excluded. After adjustment, 14 females (3 cases and 11 cancer-free) were excluded. As a result, the control group enrolled 69 females (mean age 53 years), and the CrO group contained 118 patients (mean age 54 years). The body mass index (BMI), the number of obese people, the number of triglycerides, and the amount of cholesterol did not differ significantly between the groups (*p* > 0.05). There was no difference in the number of smokers and females with hypertension (*p* > 0.05) (Table S2). Thus, the control group was matched to cases according to gender, age, body mass index (BMI), cholesterol, smoking status, the same medical center, and the same division for biochemical analyses (Table S2). In contrast to the control group, diabetic women (nine people) were identified in the CrO group. Additionally, the level of high-density lipoprotein cholesterol (HDL) was significantly higher in the control group (*p* < 0.0001).

Blood serum samples from patients diagnosed with CrO before anti-cancer treatment and from cancer-free women were analyzed in the study. 

Ninety-six percent of females in the CrO group were diagnosed with tumors of epithelial genesis, most of which (70%) were high-grade serous carcinoma (HGSC) (Table S3). All cases were microscopically verified. The tumors were staged according to the International Federation of Gynecology and Obstetrics (FIGO) classification [2,41,42]. Most patients (77%) were diagnosed with FIGO stage III or IV disease.

The study was approved by the ethical committees of the Institute of Gene Biology of the Russian Academy of Sciences (permission: 18 January 2021), the P.A. Hertsen Moscow Oncology Research Institute—branch of the National Medical Research Radiological Center of the Ministry of Health of the Russian Federation (permission: 10 December 2021), and the A. Tsyb Medical Radiological Research Center—branch of the National Medical Research Radiological Center of the Ministry of Health of the Russian Federation (permission: 26 January 2018). The study was conducted in accordance with the principles of the Declaration of Helsinki.

### 2.3. Iron Status Assessment

Iron status was determined on a Beckman Coulter AU (Brea, CA, USA) series analyzer. Transferrin and ferritin were quantified by immunoturbidimetry using the manufacturer’s reagents OSR6152 and OSR61203 (Beckman Coulter, USA), respectively [43]. The biochemical spectrum iron status included the determination of serum iron, total iron-binding capacity, transferrin saturation, ferritin, and transferrin (Table S4).

The principle of transferrin determination is based on the reaction with polyclonal goat antibodies to transferrin with the formation of insoluble complexes. The absorbance of the reaction mixture at 380 nm is proportional to the transferrin concentration in the sample. The analytical range is 0.75–7.5 g/L, and it is performed spectrophotometrically on a Beckman Coulter AU series analyzer (USA).

### 2.4. Genotyping

The Single Nucleotide Polymorphisms (SNPs) G845A rs 1800562 and C187G rs 1799945 of the *HFE* gene were genotyped in individuals with elevated TSAT (>40%) or ferritin levels of 400 µg/L or more, as we described earlier [44] (Supplemental S1).

### 2.5. Immunoaffinity Fractionation of Holo-Tf and Apo-Tf Using Single-Domain Antibodies for Holo-Transferrin (aTf1) and Apo-Transferrin (aTf2)

To isolate holo-Tf and apo-Tf, we used previously obtained immunosorbents with immobilized corresponding nanobodies, as described previously [40]. Both nanobody variants were adapted, worked up in bacterial periplasm, and purified. The adapted nanobodies (holo-specific aTf1, apo-specific aTf2, or both nanobodies together) were immobilized on CNBr-sepharose (300 µg per 100 µL of sepharose). The obtained immunosorbents were used to isolate different forms of transferrin from the blood plasma of eight patients with ovarian cancer and a number of plasma samples from cancer-free women (control). Ten μL of the original plasma was diluted 5-fold in 1xPBS and layered on a column containing 100 μL of the immunosorbent. This plasma/immunosorbent ratio ensures that all antigens (holo-Tf or apo-Tf) specifically recognized by the immobilized nanobody are bound from the test sample by a margin. Experiments with different samples were performed in parallel on separate columns. The unbound material was washed with 1xPBS, and then the bound material was eluted three times with 100 μL of 0.1 M Glycine-HCl pH2.5, followed by neutralizing the eluate by adding 1 M Tris (the final total eluate volume was 322 μL). The eluates were analyzed in 5–19% gradient SDS-polyacrylamide gel according to Lammley. Non-reducing conditions were used (for the better separation from transferrin of possible non-specific binding serum albumin). Electrophoresis was performed on a MiniProtean 3 device (Bio-Rad, Hercules, CA, USA), and the power source was Elf-4 (DNA-Technology, Russia). PageRuler Plus Prestained Protein Ladder (Thermo Fisher Scientific, Waltham, MA, USA) was used as a protein marker. The amount of transferrin in the band on the electropherogram after staining the proteins in the gel with Imperial Protein Stain (Thermo Scientific, USA) was evaluated in the Adobe Photoshop CS6 program using the ‘histogram’ tool set to the ‘color’ channel. Deviation was used as the main measurement parameter.

### 2.6. Data and Statistical Analyses

The European Society for Medical Oncology (ESMO) and the World Health Organization (WHO) recommendations were used to determine the iron status [16,25]. Numerical variable (age, transferrin, and ferritin) values are presented in the tables as the mean (Standard Deviation). The two-sided Fisher’s exact test was performed to evaluate the differences between the clinical and tumor characteristics of the patients. A two-tailed unpaired *t*-test has been applied to compare the quantitative values that follow a normal distribution. A nonparametric Mann–Whitney test was used to compare the quantitative values that did not follow a normal distribution. When comparing three or more unpaired groups, a nonparametric Kruskal–Wallis test was used. Spearman correlation analysis was used to assess the relationship between the indices and the health status. The prognostic efficacy of transferrin was assessed using receiver-operating-characteristic curve (ROC) analysis with the calculation of the area under the operating characteristic curve (AUC).

## 3. Results

### 3.1. Clinical Indicators of the Status of Iron Metabolism in the Group of Patients with Ovarian Cancer and in Women without Malignant Diseases

Data analysis revealed that iron metabolism parameters were significantly associated with malignant ovarian diseases (*p* < 0.0001), revealing a positive correlation between CrO and ferritin levels (r = 0.392) and a negative correlation between CrO and transferrin (r = −0.450). The results of the transferrin and ferritin measurements in females in the study and control groups are presented in Table 1, which also shows the distribution of patients by stage of the disease and age. The average age of patients with advanced stages is not significantly higher compared with that of sick women with the early stages of the disease (*p* = 0.09) (Table 1).

The values of iron status differ at a high level of significance (*p* < 0.0001).

The transferrin levels in women in the CrO group (mean (SD) 2.24 (0.6) g/L) were significantly lower compared to those in the control group (mean (SD) 2.79 (0.6) g/L, *p* < 0.0001). At the same time, the score in patients with stages I and II was significantly higher than that in patients with advanced disease (means (SD) 2.51 (0.6) g/L and 2.16 (0.5) g/L, respectively; *p* = 0.0002) and significantly lower than that in the control group (*p* = 0.015) (Table 1). The degree of difference with the control was even more pronounced in patients with advanced stages of tumor development (*p* < 0.0001). Iron-storing ferritin was significantly higher in the study group compared to the control (means (SD) 179 (155) µg/L and 84.1 (80.00) µg/L, respectively, *p* < 0.0001). Maximum values were observed in patients with advanced stages (means (SD) 196 (153) µg/L), which exceeded ferritin both in the control group (84.1(80) µg/L; *p* < 0.0001) and in patients with initial stages of the disease (122 (154) µg/L; *p* = 0.003) at a high level of significance. The ferritin index in patients with the initial stages of the disease was significantly higher than that in the control (*p* < 0.0001).

### 3.2. Identification of Patients with Associated Disorders of Iron Metabolism among Women with Ovarian Cancer

**Figure 1:**
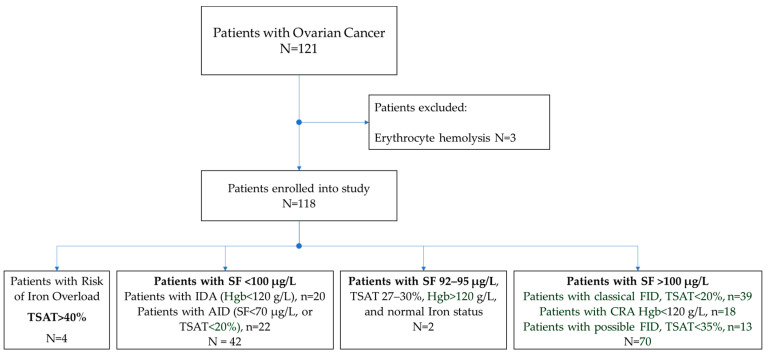
Flow diagram of CrO patients. SF—serum ferritin, TSAT—transferrin saturation, Hgm—hemoglobin, IDA—iron deficiency anemia, AID—absolute iron deficiency, FID—functional iron deficiency, CRA—cancer-related anemia.

Taking into account the recommendations of the World Health Organization (WHO) [25], the ESMO [16], the British Committee for Standards in Hematology (BSH) [45,46], the European Association for the Study of the Liver (EASL) [47], and the Royal College of Pathologists of Australasia [48], we distinguished groups of CrO patients with different values of serum ferritin, transferrin saturation (TSAT), and hemoglobin in order to identify patients with anemia and various disorders of iron metabolism (Figure 1).

Four groups of patients were formed:

1. SF < 100 μg/L. Iron-deficiency anemia (IDA): Hgb < 120 g/L, N = 20, and absolute iron-deficiency (AID): SF < 70 μg/L or TSAT < 20%, N = 22, total 42 (36%);
2. SF > 100 μg/L. Cancer-related anemia (CRA): Hgb < 120 g/L, N = 18, and patients with classical functional iron deficiency (FID): SF > 100 μg/L and TSAT < 20%, N = 39; possible FID: SF > 100 μg/L, TSAT < 35%, N = 13, total = 70 (59%);
3. Risk of iron overload: TSAT > 40%, N = 4 (3%);
4. Normal iron status: SF 92–95 μg/L, TSAT 27–30%, Hgb > 120 g/L. N = 2 (2%).

In each group, the average Tf value was calculated (Table 2). This analysis revealed that most patients had ferritin levels exceeding 100 µg/L, the vast majority of which were patients with FID (44%) and CRA (15%). Women with IDA and AID accounted for 17% and 19%, respectively, of those examined. Iron overload was observed in four women with CrO, and only in two cases could a normal iron status could be stated. The genotyping of patients with TSAT >40% (iron overload, T = 4) revealed them to be carriers of the SNPs G845A rs 1800562 or C187G rs 1799945 of the *HFE* gene, which are associated with increased iron storage (Table S5).

### 3.3. Comparison of Transferrin Levels between Patients with Different Iron Statuses and the Control Group

Reduced transferrin levels have been found to be more common among individuals with FID and CRA. The mean (SD) value of Tf in this combined group is significantly lower compared to that of CrO patients with associated AID and IDA (1.96 (0.4) g/L vs. 2.65(0.5) g/L, *p* < 0.0001) (Table 2). In addition to decreased transferrin and increased ferritin, FID + CRA in patients with ovarian cancer was associated with older age and excessive weight compared to another type of iron deficiency (AID + IDA) (Table 2). A high significance of differences between the Tf values in the two groups with associated iron deficiency (FID + CRA vs. AID + IDA) was observed at the early stages of the CrO (*p* = 0.0003). 

The proportion of individuals with reduced Tf levels (<2 g/L) in the group of individuals with FID + CRA (56%) was significantly higher (*p* < 0.0001) compared to the other category of iron deficiency (AID + IDA, 7%) associated with CrO (Table 3). At the early stages of cancer development, individuals with reduced transferrin values represented the majority among those with FID + CRA (60%). One patient with CrO out of four with elevated iron contents had a decreased Tf value (TSAT = 48.8%; SF = 301 μg/L; Tf = 1.6 g/L). Only one patient diagnosed with CrO from the IDA group had a Tf concentration above the upper threshold of normal (TSAT = 5.6%, SF = 13.8 μg/L; Tf = 3.85 g/L; HGB = 110 g/L).

**Figure 2:**
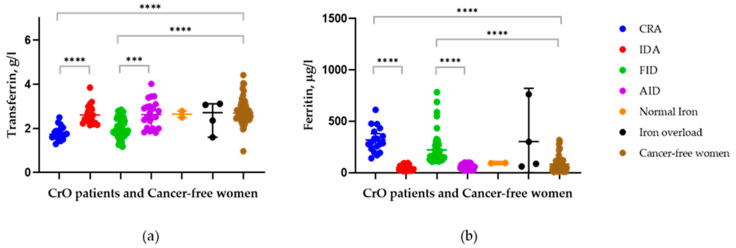
Transferrin (**a**) and ferritin (**b**) levels in groups of CrO patients with different iron statuses and in the cancer-free women (Control). Note: *** *p* < 0.001; **** *p* < 0.0001. *p*—*p*-value.

One control female had a decreased Tf value coupled with a normal iron status. Elevated Tf values in the control group were observed in three women with absolute iron deficiency (TSAT < 10%, SF < 4 μg/L, Tf > 4 g/L) and in three women with normal iron status—a total of six people (9%). The lowest Tf and the highest average SF levels were observed in individuals with CRA ((mean (SD): 1.79 (0.3) g/L and 319 (123) μg/L, respectively). Compared with FID ((mean (SD): 2.02 (0.4) g/L and 222 (145) μg/L, respectively), the differences are significant (*p* = 0.04 and *p* = 0.01) (Figure 2). 

The transferrin levels in both FID and CRA patients are significantly lower compared to the AID, IDA, normal iron status, iron overload, and control (Table 4 and Table 5). 

The most pronounced differences between Tf levels for both the CRA and FID groups were observed in patients with associated AID (*p* < 0.0001) or IDA (*p* < 0.0001) and in those in the control group (*p* < 0.0001) (Figure 2, Table 5). To a lesser extent, but at significant levels, differences in the Tf level for both CRA and FID were found in groups with iron overload (*p* = 0.003 and *p* = 0.03, respectively) and a normal iron status (*p* = 0.002 and *p* = 0.04, respectively). One CrO patient with IDA (TSAT: 5.64%; SF: 13.8 μg/L; HGB: 110 g/L) had an elevated level of Tf (3.85 g/L). Patients with associated FID or CRA have high SF levels (mean (SD): 222 (145) μg/L and 319 (123) μg/L), respectively, but it did not differ significantly among patients with iron overload status (mean (SD): 303 (325) μg/L) (*p* > 0.05)) (Table 4 and Table 5). According to our data, Tf is more informative than SF in the identification of CRA or FID among CrO patients with associated different types of impaired iron homeostasis. Table 4 shows the TSAT values in different groups of patients with ovarian cancer, as well as the significance levels of the differences. The differences between the two types of anemia (*p* = 0.574) and the two types of iron deficiency (*p* = 0.695) are not significant, i.e., the main limitation of TSAT is the inability to distinguish FID from AID and CRA from IDA in patients with CrO. Table 5 clearly demonstrates the advantage of Tf values over TSAT in determining the type of iron deficiency: CRA vs. IDA for Tf *p* = 0.0001 and for TSAT *p* = 0.584; FID vs. AID for Tf *p* = 0.0001 and for TSAT *p* = 0.695. 

### 3.4. Analysis of the Ratio of Holo- and Apo- Forms of Transferrin in Cancer Patients and the Control

**Figure 3:**
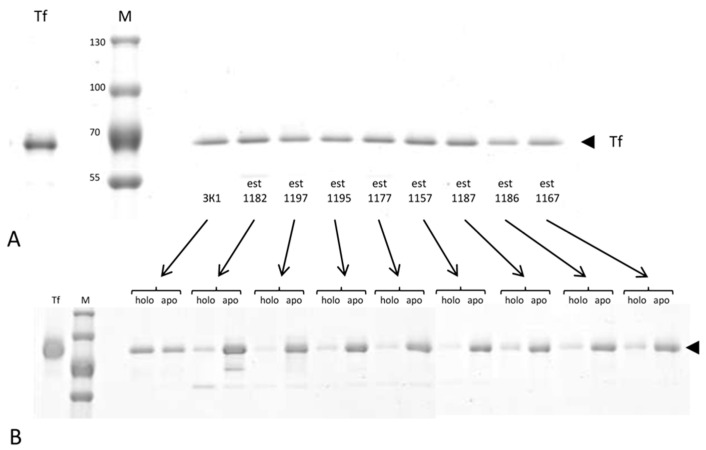
Demonstration of a marked decrease in the proportion of iron-saturated transferrin (holo-Tf) relative to iron-free transferrin (apo-Tf) in the blood of CrO patients using a method based on immunoaffinity chromatography followed by the electrophoretic fractionation of specifically bound and then eluted transferrin isoforms. M is a protein marker; marker protein sizes in kDa are indicated. Tf is a commercial preparation of transferrin. Gradient: 5–19% SDS-polyacrylamide gel. (**A**) immunosorbents—both antibodies simultaneously; (**B**) two separate immunosorbents: aTf1 (anti-holo-Tf, holo) and aTf2 (anti-apo-Tf, apo). Markers: est—CrO patients, 3K1—control.

Using immunosorbents based on single-domain antibodies to holo-transferrin (aTf1) and apo-transferrin (aTf2) for the immunoaffinity fractionation of holo-Tf and apo-Tf, we performed a direct analysis of the ratio of these transferrin forms in a series of CrO patients and control women (Table 6). Figure 3 shows results of the combined assay for a small number of blood plasma samples.

Single-domain antibodies were used as ligands in the immunosorbents: either both antibodies simultaneously (Figure 3A) or separately (Figure 3B) were used—aTf1 (anti-holo-Tf, holo) and aTf2 (anti-apo-Tf, apo). The arrow on the right indicates the position of transferrin on electropherograms. The upper part of the figure (Figure 3A) shows the results of the isolation of total transferrin using an immunosorbent containing both variants of nanoparticles.

## 4. Discussion

The results obtained in the present study are in accordance with the data described in the literature on transferrin and ferritin as negative and positive reactants of the acute phase of inflammation, respectively [12,31,49]. During the development of cancer, the ferritin concentration increases, and the transferrin concentration decreases, often regardless of iron status, and iron deficiency can be diagnosed with ferritin values exceeding 50 µg/L [50,51]. In our study, the reference interval of Tf values (2–3.6 g/L) was established in 73 CrO patients, 2 patients were found to have elevated transferrin levels, and 43 (36%) individuals were determined to have reduced transferrin values.

We calculated AUC values using operating characteristic curve analysis (ROC-curves) to compare the effectiveness of ferritin and transferrin in predicting CrO risk.

The AUC values for transferrin and ferritin were 0.769 (95%CI (0.702–0.827), *p* < 0.0001) and 0.738 (95%CI (0.669–0.800), *p* < 0.0001), respectively, without significant difference between them (*p* = 0.431). Based on the values obtained, transferrin can be considered as a good classifier of CrO. For ferritin, the marker is an elevated level; at the cutoff point ≥ 85.1 μg/L, the sensitivity of the test is 70.34% 95%CI (61.2–78.4), and the specificity is 73.91% 95%CI (61.9–83.7). For transferrin, a reduced level serves as a marker of CrO; at the cut-off point, a concentration of ≤2.36 g/L corresponds to a sensitivity of 60.17% 95%CI (50.7–69.1) and a specificity of 86.96% 95%CI (76.7–93.9). A decrease of the transferrin level increases specificity. Thus, at transferrin concentrations ≤ 1.95 g/L, the specificity is 98.55% 95% (92.2–100), but the sensitivity is low at 38.98% 95%CI (30.1–48.4). The specificity of transferrin is higher than that of ferritin. In a small study (37 CrO patients, 31 females with benign ovarian disease, and 31 control females) the prognostic properties of Tf in assessing the risk of CrO were also analyzed [52]. At the cut-off point (<2.3 g/L), the sensitivity (73%) and specificity (74%) are almost equal to each other. We can assume that with a larger sample of patients and control individuals, these values would have been different. Apart from this, the status of iron was not analyzed by the authors.

The distribution of female patients into the IDA + AID and FID + CRA groups, which differ from each other in terms of ferritin indices, revealed a predominant majority of patients with low transferrin in the FID + CRA group (N = 39, 91%). Three women with reduced Tf levels were assigned to the AID group according to ESMO recommendations because their SF was below 100 μg/L (SF between 84.2 and 96.5 μg/L) and their TSAT was below 20% [16]. Taking into account that Tf is a negative reactant of the acute phase of inflammation, ferritin values are close to 100 μg/L, and the TSAT is low, it is most likely that these three females have ovarian cancer associated with FID. In a malignant disease, interpreting the type of iron deficiency on the basis of ferritin concentrations and TSAT is not an easy task. International guidelines for the diagnosis of iron deficiency in chronic inflammation are heterogeneous, and, depending on the disease, the threshold values of ferritin and TSAT differ [50]. In the absence of inflammation and cancer, ferritin levels less than 30 µg/L are the most specific test for detecting iron deficiency in the body [25]. The WHO guidelines suggest a lower threshold for ferritin (<70 µg/L) to determine iron deficiency in patients affected by inflammatory diseases [25]. However, it has been shown that patients with malignant diseases often have ferritin values as high as 100 µg/L despite the absence of staining iron in the bone marrow (absolute iron deficiency), the SF reference interval can indicate both low and normal iron stores, and ferritin values above the normal range correspond to both normal and excessive iron stores [53]. It was suggested to diagnose FID based on the cumulative normal values range of TSAT, SF, and hemoglobin values [24]. In a special study on the determination of iron deficiency status in patients with diseases accompanied by chronic inflammation, a criterion was proposed for cancer patients that corresponded to ferritin levels less than 100 µg/L and/or TSAT levels less than 20% for the AID [49]. Another recommended criterion for the diagnosis of FID is a ferritin level > 100 µg/L combined with low transferrin saturation (TSAT < 20%) [16,54] or with normal TSAT (20–40%) [32,46,53]. TSAT has its limitations, showing inadequately high or false normal values [16], and at the early stages of iron deficiency, it is often within the reference level [32]. Ferritin levels, in turn, are elevated not only in cases of cancer and inflammation but also in metabolic syndrome, diabetes, chronic alcoholism, cytolysis, and hemochromatosis, caused mainly by polymorphisms (SNP) of the *HFE* (High Fe 2+) gene (Cys282Tyr rs 1800562 and His63Asp rs 1799945) [55,56]. Therefore, in the range of SF values from 70 µg/L to 100 µg/L, it is difficult to distinguish the type of iron deficiency associated with malignant disease. In our study, this transition group between absolute iron deficiency and functional iron deficiency consisted of 10 people. So, ferritin should not be used alone to diagnose iron status [57]. Based on the analysis of our data, we have shown that reduced Tf values together with low TSAT reveals FID associated with CrO. Differences in the Tf value between the two types of iron deficiency and the two types of anemia (also between FID + CRA and AID + IDA) are at a high level of significance (*p* < 0.0001). In the case of FID and CRA, the level of Tf is significantly lower compared to AID and IDA. Since transferrin is an acute-phase protein, its concentration decreases in response to cytokine production, and in contrast to the FID, absolute iron deficiency is diagnosed when transferrin values are normal and high. The pathogenesis of CRA and FID is not considered to be based on iron deficiency per se but on the disruption of iron homeostasis due to chronic inflammation and oxidative stress [19]. 

In screening for iron overload conditions, the TSAT is often preferred because it is elevated with little iron overload [30,32,47,53]. A suggested indicator of iron overload is an SF above 150 µg/L for menstruating women and above 200 µg/L for menopausal women and TSAT above 40% or 45%, respectively [48]. In our study, all four iron overload patients (TSAT > 44%) were carriers of one of these two SNP *HFE* genes (Table S5). 

Thus, data analysis showed that low transferrin levels in females with ovarian cancer are associated with functional iron deficiency, CRA and advanced stages of the disease.

To evaluate the prognostic value of transferrin as a marker of functional iron deficiency in malignancy, we calculated the AUC, sensitivity, and specificity of the test using ROC analysis. Taking 2.11 g/L as the cutoff point, the sensitivity was 71.62% 95%CI (59.9–81.5), the specificity was 93.18% 95%CI (81.3–98.6), and the AUC was 0.896 95% CI (0.826–0.944), *p* < 0.0001. The high AUC of 0.896 indicates that transferrin has a high potential to assess the risk of functional iron deficiency in patients diagnosed with ovarian cancer, allowing for the detection functional iron deficiency even in asymptomatic patients at the initial stages of the disease, which is a signal of the onset of malignant growth and is likely to lead to the development of CRA.

CRA greatly complicates antitumor therapy and is a poor prognostic factor. Currently, there is no single reliable and widely available biochemical marker of functional iron deficiency [12]. Transferrin, in our study, showed a high potential as a biomarker of functional iron deficiency and CRA.

Routine laboratory tests of plasma iron status (determination of serum iron, total iron-binding capacity, ferritin, transferrin, and transferrin saturation) are rather general characteristics that can miss more subtle changes in transferrin forms in different biological processes and fluids. To investigate such likely changes in transferrin forms, methods based on the use of highly specific monoclonal antibodies for specific forms of transferrin may be more adequate tools than polyclonal antibodies. We have recently obtained such single-domain antibodies for different forms of transferrin using a technique that we have been developing and using in many studies [40,58,59,60,61]. The new single-domain antibodies for holo-transferrin (aTf1) and apo-transferrin (aTf2), as well as immunosorbents based on them, differentially bind various iron-saturated forms of transferrin. This different binding allows us to observe changes in the representation of various forms of transferrin associated directly or indirectly with cancer in a number of patients compared with cancer-free women. In this study, we used similar immunosorbents and more carefully selected immunoaffinity fractionation conditions to directly analyze the ratio of holo-Tf to apo-Tf in a number of CrO patients. 

The immunosorbent for the isolation of total transferrin allows for a highly specific isolation of transferrin and the estimation of its visible amount directly in the corresponding gel band (Figure 3A). Note that the observed variations in the amount of total transferrin for the samples taken are relatively small. In our opinion, we can see a more obvious differentiating effect associated with a marked decrease in the proportion of iron-saturated transferrin (holo-Tf) relative to iron-free transferrin (apo-Tf) in the blood of patients with advanced-stage CrO using a method based on immunoaffinity chromatography followed by the electrophoretic fractionation of transferrin isoforms specifically bound and then eluted (Figure 3B). Although, normally, the holo-Tf/apo-Tf ratio was determined in the range of 30–55%/45–70% (Figure 3 shows the result for only one cancer-free woman’s sample, 3K1, with the highest holoTf content), in CrO, this ratio is 10–23%/77–95% (demonstrated for eight samples from patients with CrO). We used immunosorbents with immobilized single-domain antibodies taken with a reserve to fully bind all the specific transferrin variants from the blood plasma aliquot taken. The results clearly demonstrate the perspectivity of this method of analysis for the identification of women with ovarian cancer.

### Limitations

Our study has several limitations. The main limitation is the relatively small number of patients and control group individuals. Secondly, the bone marrow iron and liver iron content, the hepcidin and serum transferrin receptor concentration, and the Hb in reticulocytes or RetHe were not examined to determine iron status. This could allow for a more accurate determination of the type of iron deficiency in patients with ferritin levels in the range of 70 to 100 μg/L. According to ESMO, WHO, and BSH recommendations, we used SF and TSAT to determine iron status in the body. Thirdly, only one plasma sample from a cancer-free female was included in the analysis of the ratio of holo- and apo- forms of transferrin. In addition, there were no samples from patients with stage I ovarian cancer and from individuals with benign ovarian disease. A comparative analysis of such samples would make it possible to assess the specificity of the ratio of holo- and apo- forms of serum transferrin. In the future, such a study should be conducted, but for now, we have shown the potential efficiency of the analysis of the holo-Tf and apo-Tf ratio using monoclonal nanoantibodies for the identification of women with ovarian cancer.

## 5. Conclusions

Low serum transferrin has been associated with malignant ovarian disease. Functional iron deficiency in patients diagnosed with ovarian cancer is associated with decreased serum transferrin levels. Transferrin has a great potential in assessing the risk of both ovarian cancer and functional iron deficiency in patients diagnosed with ovarian cancer. We suggest that the use of immunoaffinity chromatography to separate holo-Tf/apo-Tf and determine their ratio can be a valuable adjunct when forming a risk group among patients with benign ovarian diseases.

## Figures and Tables

**Table 1:** Indicators of iron status in patients with ovarian cancer and those in the control group.

Parameters	Control N = 69	CrO N = 118	*p* Value	Patients with Ovarian Cancer Stages I–II N = 27	Patients with Ovarian Cancer Stages III–IV N = 91	*p* Value
Age, years Mean (SD)	53(10.5)	54(11.8)	0.619	48(15.2)	55.5(10.1)	0.09
Iron, μmol/L Mean (SD)	16.8(6.1)	8.58(5.6)	<0.0001	9.72(5.0) **	8.24(5.8) *	0.01
TSAT, % Mean (SD)	28.0(10.9)	16.4(9.0)	<0.0001	17(8.0)	16.2(9.3) *	0.31
Transferrin, g/L Mean (SD)	2.79(0.6)	2.24(0.6)	<0.0001	2.51(0.6) **	2.16(0.5) *	0.0002
Ferritin, μkg/L Mean (SD)	84.1(80.00)	179(155)	<0.0001	122(154) *	196(153) *	0.003
Hemoglobin, g/L Mean (SD)	135(10.1)	123(14.0)	<0.0001	127(14.7)	122(13.8) *	0.09
N/A, N	0	11	0.0008 ^‡^	5	6	0.123 ^‡^

Note: * *p* < 0.0001, ** *p* < 0.02, compared to control. *p*—Unpaired *t*-test. ‡—Two-sided Fisher’s exact test. N/A—not available. TSAT—transferrin saturation.

**Table 2:** Transferrin values in ovarian cancer patients with different iron statuses.

Parameter	AID + IDA N = 16	FID + CRA N = 10	*p*	AID + IDA N = 26	FID + CRA N = 60	*p*	AID + IDA N = 42	FID + CRA N = 70	*p*	Norm + Iron Over-Load N = 6
	I + II Stage			III + IV Stage			Total			
Age, years	43.1(15.2)	58.1(10.1)	0.011	51.9(9.03)	57.4(9.9)	0.018	48.5(12.4)	57.5(9.9)	<0.0001	49(14.7)
BMI, kg/m^2^	26.2(4.4)	27.6(4.0)	0.414	27.7(5.0)	29.5(5.4) *	0.151	27.12(4.8)	29.2(5.3) *	0.036	28.7(7.0)
Tf, g/L	2.78(0.4)	2.05(0.4) **	0.0003	2.57(0.6)	1.95(0.4) ***	<0.0001	2.65(0.5)	1.96(0.4) ***	<0.0001	2.57(0.6)

Note: * *p* < 0.05; ** *p* < 0.001; *** *p* < 0.0001, compared to the control group. *p*—*p*-value. AID—absolute iron deficiency; IDA—iron deficiency anemia; CRA—cancer-related anemia; BMI—body mass index, Tf—transferrin. Data presented as the mean (SD).

**Table 3:** The distribution of patients with a low transferrin (<2 g/L) concentration in ovarian cancer patients with different iron statuses and the control group.

Parameter	AID+ IDA N = 16	FID+ CRA N = 10	*p* Value	AID+ IDA N = 26	FID+ CRA N = 60	*p* Value	AID+ IDA N = 42	FID+ CRA N = 70	*p* Value	Normal Iron N = 2	Iron Overload N = 4	Total CrO N = 118	Control N = 69	*p* Value
	I+II Stage			III+IV Stage			Total							
Tf low N (%)	0	6 (60)	0.002	3 (12)	33 (55)	0.0001	3 (7)	39 (56)	<0.0001	0	1 (25)	43 (36)	2 (3)	<0.0001
Tf > 2g/L N (%)	16 (100)	4 (40)		23 (78)	27 (45)		39 (93)	31 (44)		2 (100)	3 (75)	75 (64)	67 (97)	

Note: AID—absolute iron deficiency; IDA—iron deficiency anemia; CRA—cancer-related anemia; FID—functional iron deficiency; Tf—transferrin.

**Table 4:** Indicators of transferrin, serum ferritin, transferrin saturation, and hemoglobin across different types of iron status in ovarian cancer patients.

Parameter	Iron Status in CrO Patients									Control N = 69
	CRA N = 18	IDA N = 20	*p* Value	FID N = 52	AID N = 22	*p* Value	Normal Status N = 2	Iron Overload N = 4	*p* Value	
Age, mean (SD) years	60 (10) *	46 (11) *	0.0002	57 (10)	51 (13)	0.045	49 (20)	49 (15)	0.96	53 (11)
BMI, mean (SD) kg/m^2^	30 (6)	28 (6)	0.282	29 (5)	27 (4)	0.049	34 (4)	26 (7)	0.275	27 (5)
Tf, mean (SD) g/L	1.79 (0.3) ***	2.64 (0.4)	0.0001	2.02 (0.4) ***	2.66 (0.6)	0.0001	2.65 (0.2)	2.54 (0.7)	0.853	2.79 (0.5)
SF, mean (SD) μkg/L	319 (123) ***	51.5 (29)	0.0001	222 (145) ***	64.7 (27)	0.0001	94 (7)	303 (325) ***	0.440	84.1 (80)
TSAT, mean (SD) %	14 (6) ***	12 (7) ***	0.584	16 (7) ***	17 (8) ***	0.695	28 (2)	47 (3) **	0.002	28 (11)
HGB, mean (SD) g/L	105 (11) ***	111 (6) ***	0.05	132 (8)	131 (8)	0.708	127 (5)	130 (7)	0.560	135 (10)

Note: * *p* < 0.01; ** *p* < 0.001; *** *p* < 0.0001, compared to the control. AID—absolute iron deficiency; IDA—iron deficiency anemia; CRA—cancer-related anemia; FID—functional iron deficiency; BMI—body mass index; Tf—transferrin; SF—serum ferritin; HGB—hemoglobin; CrO—cancer ovary.

**Table 5:** Significance levels of differences (*p*-value) in transferrin, serum ferritin, and transferrin saturation values between patients with FID or CRA and patients with other types of iron homeostasis and the control.

Parameter	Tf	SF	TSAT	Parameter	Tf	SF	TSAT
CRA				FID			
Control	0.0001	0.0001	0.0001	Control	0.0001	0.0001	0.0001
FID	0.043	0.01	0.184	CRA	0.043	0.01	0.043
IDA	0.0001	0.0001	0.584	IDA	0.0001	0.0001	0.054
AID	0.0001	0.0001	0.159	AID	0.0001	0.0001	0.695
Iron overload	0.003	0.861	0.0001	Iron overload	0.03	0.336	0.0001
Normal iron status	0.002	0.021	0.002	Normal iron status	0.04	0.223	0.015

Note. AID—absolute iron deficiency; IDA—iron deficiency anemia; CRA—cancer-related anemia; FID—functional iron deficiency. Tf—transferrin; SF—serum ferritin; TSAT—transferrin saturation.

**Table 6:** The serum ratio of holo-transferrin to apo-transferrin in the study group and the control group, as determined by immunoaffinity chromatography.

Patient Code	Age, Years	Diagnosis	TNM	Ratio Holo/Apo Tf
3K1	60	Control	-	55.65%/44.35%
Est1182	57	HGSC	T3cN0M1	18.76%/81.24%
Est1197	63	HGSC	T3cN0M1	12.59%/87.41%
Est1195	69	HGSC	T3cN0M1	16.33%/83.67%
Est1177	34	HGSC	T2bN0M1	17.44%/82.56%
Est1157	57	HGSC	T3cN0M1	10.04%/89.96%
Est1187	61	HGSC	T3cN0M1	21.92%/78.08%
Est1186	56	HGSC	T3cN0M1	19.49%/80.51%
Est1167	70	LGSC	T3cN0M0	23.32%/76.68%

Note: HGSC—high-grade serous carcinoma; LGSC—low-grade serous carcinoma; Tf—transferrin.

## Data Availability

There were no publicly archived datasets analyzed or generated during the study.

## Supplementary Materials

The following supporting information can be downloaded at: https://www.mdpi.com/article/10.3390/jcm11247377/s1, Table S1. Data collected from the clinical records of the patients enrolled in the study, either cases or controls; Table S2. Clinical characteristics, comorbidities in patients and cancer-free women; Table S3. Clinical characteristics of patients in accordance with the International Federation of Gynecology and Obstetrics (FIGO) classification; Table S4. Laboratory findings of iron status at admission; Table S5. Data on the genotyping of the *HFE* gene in individuals with iron overload. Supplemental S1. Genotyping the SNPs G845A rs 1800562 and C187G rs 1799945 of the *HFE*.
