# Supplementary material for: Prognostic Value of Serum Transferrin Analysis in Patients with Ovarian Cancer and Cancer-Related Functional Iron Deficiency: A Retrospective Case–Control Study
Source: J Clin Med. 2022 Dec 12;11(24):7377. doi: 10.3390/jcm11247377 (PMC9786287; doi:10.3390/jcm11247377)
Supplement: Supplementary file 1 [file jcm-11-07377-s001.zip › jcm-2066629-supplementary.pdf]

Table S1. Data collected from the clinical records of the patients enrolled in the study, either cases or controls.

**Data collected from the clinical records of the patients enrolled in the study, either cases or controls.**
Age
BMI
Date of biochemical and haematologic analysis at admission
Patient and control comorbidities, including smoking, hypertension, diabetes, renal failure, chronic liver failure, solid or blood cancer, lipid profile.
Previous hospitalization, previous administration of a broad-spectrum antibiotic

Table S2. Clinical characteristics, comorbidities in patients and controls.

**Characteristics**	**Controls N=69**	**CrO N=118**	**P value**	**Patients with Ovarian Cancer Stages I-II N=27**	**Patients with Ovarian Cancer Stages III-IV N=91**	**P value**
Age, years Mean (SD)	53(10.5)	54(11.8)	0.619	48(15.2)	55.5(10.1)	0.09
BMI, kg/m2 Mean (SD)	27(5.1)	28.5(5.2)	0.07	27(4.6)	28.9(5.4)	0.02
Exposure unknown, N(%)	3(4)	0	0.05‡	0	0	-
BMI≥30 kg/m2 N(%)	16(23)	41(35)	0.103‡	8(30)	32(35)	0.650
N/A, N	0	11	0.0008‡	5	6	0.123‡
Triglycerides, mmol/L Mean (SD)	1.35(0.6)	1.48(0.6)	0.08	1.36(0.6)	1.51(0.7)	0.873
HDL cholesterol, mmol/L Mean (SD)	1.52(0.3)	1.27(0.3)	<0,0001	1.36(0.4)	1.24(0.3) **	0.363
Cholesterol, mmol/L Mean (SD)	5.78(1.1)	5.92(1.1)	0.26	5.72(1.2)	5.79(1.1)	0.934
Smoking,N	7	9	0.594‡	1	8	0.68‡
Exposure unknown, N	2	4	>0.999‡	1	3	>0.99‡
Diabet, N	0	9	0.03‡	2	7*	>0.99‡
Exposure unknown, N	5	5	0.50‡	2	3	0.32‡
Hypertension, N	17	44	0.13‡	6	38	0.10‡
Exposure unknown, N	12	6	0.01‡	2	4 **	0.62‡
Up to 48 years old, N	21	30	0.498‡	12	18	0.02‡

Note: * P<0,05; ** P<0,02 compared to control. P – Unpaired t-test. ‡Two-sided Fisher's exact test.

Table S3: Clinical characteristics of cohort patients in accordance with the International Federation of Gynecology and Obstetrics (FIGO) classification [Prat J; FIGO Committee on Gynecologic Oncology. Staging classification for cancer of the ovary, fallopian tube, and peritoneum. Int J Gynaecol Obstet. 2014 Jan;124(1):1-5. doi: 10.1016/j.ijgo.2013.10.001],

[Mutch DG, Prat J. 2014 FIGO staging for ovarian, fallopian tube and peritoneal cancer. Gynecol Oncol. 2014 Jun;133(3):401-4. doi: 10.1016/j.ygyno.2014.04.013.]

**Clinical characteristics of patients in accordance with the International Federation of Gynecology and Obstetrics (FIGO) classification**	
**Characteristics**	**CrO N=118 N(%)**
**Histologic types**	
High-grade serous carcinoma (HGSC)	83 (70)
Low-grade serous carcinoma (LGSC)	5 (4)
Mucinous carcinoma (MC)	3(2)
Endometrioid carcinoma (EC)	10(9)
Clear-cell carcinoma (CCC)	10(9)
Malignant germ cell tumors	2(2)
Cannot be classified	5(4)
**FIGO stages**	
I	17(14)
II	10(9)
III	61(52)
IV	30(25)

Table S4. Laboratory findings of iron status at admission.

**Laboratory findings of iron status at admission.**
Serum iron
Total iron binding capacity
Transferrin Saturation (TSAT)
Serum Transferrin
Serum Ferritin
Hemoglobin

Supplemental S1.

Genotyping SNPs G845A rs 1800562 and C187G rs 1799945 of the *HFE*.

Single Nucleotide Polymorphisms (SNPs) G845A rs 1800562 and C187G rs 1799945 of the *HFE* gene were genotyped in individuals with elevated TSAT (>40%) or ferritin of 400 µg/L or more as we described earlier (Ivanova TI, 2015). DNA was extracted from 300 µL of whole blood using Wizard Genomic DNA Purification Kit (Promega, Madison, WI, USA). Genotypes were determined by a PCR-based restriction fragment length polymorphism (RFLP) technique. The 389-bp fragment of the *HFE* gene including codon Cys282Tyr was amplified with primers F 5'- TGGCAAGGGTAAACAGATCC and R 5'-CTCAGGCACTCCTCTCAACC; the 208-bp fragment of the *HFE* gene containing codon His63Asp was amplified using primers F 5'-ACATGGTTAAGGCCTGTTGC and R 5'- GCCACATCTGGCTTGAAATT. The fragments were amplified in 25-µL reaction volume containing 0,625 U of HotStarTag polymerase (Quiagen, Valencia,USA), 0,15 mM dNTPs, 0,2 µM primers, and 50 ng of genomic DNA. PCR-reactions were carried out as follows: at first 15 min denaturing at 95 °C, and then 30 cycles (30 s at 94 °C, 1 min at 60 °C, 30 s at 72 °C) for *HFE* and final 10 min extension at 72 °C. Amplified fragments were digested with either Rsa I (Promega, Madison, WI, USA) or BclI (SIGMA, Saint Louis, MO, USA) for detection of the Cys282Tyr or His63Asp of the *HFE* gene.

Table S5. Data on genotyping of the *HFE* gene in individuals with iron overload.

**Data on genotyping of the *HFE* gene in individuals with iron overload**					
Patients code	Age	G845A rs 1800562	Status G845A	C187G rs 1799945	Status C187G
Est1177	35	GG	wild types	GG	homozygous
Est1202	70	GA	heterozygous	CC	wild types
Est1218	47	GG	wild types	CG	heterozygous
Est1225	45	GG	wild types	CG	heterozygous
